# Genome-Wide Identification of Bone Metastasis-Related MicroRNAs in Lung Adenocarcinoma by High-Throughput Sequencing

**DOI:** 10.1371/journal.pone.0061212

**Published:** 2013-04-08

**Authors:** Lin Xie, Zuozhang Yang, Guoqi Li, Lida Shen, Xudong Xiang, Xuefeng Liu, Da Xu, Lei Xu, Yanjin Chen, Zhao Tian, Xin Chen

**Affiliations:** 1 Department of Medical Oncology, The Third Affiliated Hospital of Kunming Medical University, Tumor Hospital of Yunnan Province, Kunming, P.R. China; 2 Department of Orthopedics, The Third Affiliated Hospital of Kunming Medical University, Tumor Hospital of Yunnan Province, Kunming, P.R. China; 3 Department of Thoracic Surgery, The Third Affiliated Hospital of Kunming Medical University, Tumor Hospital of Yunnan Province, Kunming, P.R. China; Queen Elizabeth Hospital, Hong Kong

## Abstract

**Background:**

MicroRNAs (miRNAs) are a class of small noncoding RNAs that regulate gene expression at the post-transcriptional level. They participate in a wide variety of biological processes, including apoptosis, proliferation and metastasis. The aberrant expression of miRNAs has been found to play an important role in many cancers.

**Results:**

To understand the roles of miRNAs in the bone metastasis of lung adenocarcinoma, we constructed two small RNA libraries from blood of lung adenocarcinoma patients with and without bone metastasis. High-throughput sequencing combined with differential expression analysis identified that 7 microRNAs were down-regulated and 21 microRNAs were up-regulated in lung adenocarcinoma with bone metastasis. A total of 797 target genes of the differentially expressed microRNAs were identified using a bioinformatics approach. Functional annotation analysis indicated that a number of pathways might be involved in bone metastasis, survival of the primary origin and metastatic angiogenesis of lung adenocarcinoma. These include the MAPK, Wnt, and NF-kappaB signaling pathways, as well as pathways involving the matrix metalloproteinase, cytoskeletal protein and angiogenesis factors.

**Conclusions:**

This study provides some insights into the molecular mechanisms that underlie lung adenocarcinoma development, thereby aiding the diagnosis and treatment of the disease.

## Introduction

Lung adenocarcinoma is the leading cause of cancer-related mortality worldwide. More than 1.2 million people were newly diagnosed with lung adenocarcinoma, and the disease causes one million deaths each year. The mortality rates have increased over the past few years [1]. The high mortality rate of lung adenocarcinoma is largely attributed to the fact that the early stages of the disease are asymptomatic and thus difficult to diagnose. In most cases, the disease has become quite serious and may have already resulted in extrapulmonary metastases by the time symptoms arise. Most deaths are not a result of the primary lung adenocarcinoma but of other metastatic tumors. Bone metastasis is one of the early and most commonly observed phenomena in lung adenocarcinoma. The bone metastasis rate of lung adenocarcinoma is as high as 22%–64% [2], [3]. The common clinical symptoms of bone metastasis include the pain in different degrees, pathological fracture, spinal cord compression and Hypercalcemia, all of which are known as the skeletal related events (SREs) [4]. Bone metastasis is the most important factor in reducing the quality of life and the chances of survival of patients of lung adenocarcinoma.

The main component of bone is mineral substance, which brings severe micro-environment to tumor cells; this suggests that metastatic tumor cells have the characteristics of local microenvironment transformation. Bone metastasis is a complex process that involves a series of molecular events involving multiple steps, factors and genes [5]. First, tumor cells separate from the primary tumor origin and then intrude into the circulatory system through newly formed tumor vessels. Most of these tumor cells are killed by the immune system and the physical pressure from the blood flow of the host. Only a very small number of the surviving tumor cells can enter into the sinusoids of the marrow cavity to invade the marrow stroma through the sinusoids' wall. The complex interactions between cancer cells and the local microenvironment of bony tissue lead to the proliferation and differentiation of cancer cells, causing the destruction of local bony microenvironment and the formation of bone metastases. In recent years, several studies have characterized the molecular biology of tumor metastases. As a result, many genes have been identified and been demonstrated to be useful as prognostic molecular biomarkers [6]. However, more efforts are needed to illustrate the molecular mechanism of lung adenocarcinoma bone metastasis to develop less invasive and more effective medical treatments.

MicroRNAs (miRNAs) are a class of small noncoding RNAs that regulate gene expression at the post-transcriptional level [7], [8]. miRNA targeting is achieved by bind to the complementary sites in the 3′-untranslated region (3′-UTR) of messenger RNAs [9]. Gene expression regulation mediated by miRNAs is widespread in mammals; more than 1,500 miRNA genes have been identified in the human genome (miRBase release 18.0) [10], and it is estimated that one-third of the genes are regulated by miRNAs [11]. miRNAs play key regulatory roles in cellular processes including apoptosis, proliferation and metastasis [12]. The aberrant expression of miRNAs is reported to involved in Burkitt's lymphomas, B cell chronic lymphocytic leukemia (CLL) and many other solid cancer types, including breast, liver, ovarian, colorectal and prostate cancer [13]. Recent analysis also revealed a number of miRNAs that have abbreviate expression pattern in the lung cancer [14]. However, the roles of miRNA played in bone metastasis of lung adenocarcinoma remains largely unknown.

Recent studies indicated that miRNAs can be released from tumor cells into the circulatory system and remain stable in the blood [15], [16]. These findings suggest that circulating miRNAs can be used as biomarkers for tumor diagnosis and prognosis. Genome-wide expression analysis of miRNA from serum has been used to predict the survival of non-small-cell lung cancer (NSCLC) patients [17]. In this study, we constructed two small RNA (sRNA) libraries from blood samples of patients of lung adenocarcinoma with and without bone metastasis. Solexa technology was then used to sequence the two libraries and detect the genome-wide changes in miRNA expression. We discovered 28 miRNAs that are differentially expressed in the samples of lung adenocarcinoma with and without bone metastasis. Functional annotation analysis of the target genes of these differentially expressed miRNAs indicated that many of them are over-represented in the MAPK, Wnt and NF-KappaB signaling pathways, as well as other cancer related pathways related to the bone metastasis of lung adenocarcinoma. This study provides improved understanding of the mechanism of bone metastasis of lung adenocarcinoma.

## Materials and Methods

### Patients and samples

Blood samples were obtained from patients of lung adenocarcinoma who hospitalized at the Tumor Hospital of Yunnan Province (The Third Affiliated Hospital of Kunming Medical University, Kunming, Yunnan, China) between January and May in 2012. The average age of the patients is 59.2 years, 21 patients are male and 19 patients are female. Among the 40 patients included, 20 are patients with bone metastasis and 20 are without bone metastasis. The patients selected for analysis were as follows: First, primitive lung adenocarcinoma were diagnosed by cytological and histological examination to exclude the second primary tumors. Second, blood samples were only collected from patients without any antineoplastic therapy. Third, patients were chosen by a careful whole body X-ray, CT, SPECT, MRI and PET-CT inspection to diagnose as primary lung adenocarcinoma with or without bone metastasis. The criteria for bone metastases diagnosis are as follows: SPECT inspection indicated the multiple metastasis and then confirmed by one of the methods, which include CT, MRI or X-ray inspection, or patients have other clinical symptoms of bone metastases, which include the local pain or tenderness, pathological fracture or paraplegia, or PET-CT indicated the bone metastasis. The patients who have primary origin and the bone metastasis were confirmed by the cytological or the histological diagnosis. Patients with the following characteristics were excluded: that have traumatic fracture in one year time before hospitalized, or have other endocrine disease could influence bone metabolism. Patients without bone metastasis were inspected by X-ray, CT, MRI, SPECT or PET-CT monthly to eliminate micrometastasis in that the metastasis were not found at least for two months. All patients were provided informed consents. Blood samples from each group were treated and then mixed to two specimens respectively for further analysis.

### RNA extraction and construction of sRNA libraries

Tumor samples were obtained from patients of lung adenocarcinoma with and without bone metastasis and frozen in liquid nitrogen. Total RNA was extracted from lysed samples using the Trizol reagent from Invitrogen (Invitrogen, Carlsbad, CA, USA) according to the provided protocols. The RNA was separated by gel electrophoresis on a 15% TBE urea denaturing PAGE gel. The quality of the total RNA was assessed by measuring the OD value. sRNAs corresponding to 18–30 nt were excised and recovered. The 18–30 nt small RNAs were adapter-ligated using T4 RNA ligase and were subsequently reverse transcribed into cDNA by Superscript II Reverse Transcriptase. The cDNA fragments were PCR-amplified for subsequent sequencing.

### Computational analysis of sRNAs

The Illumina HiSeq sequencer was used to generate raw sequence reads. Raw sequencing data for the transcriptome have been deposited in the DDBJ Sequence Read Archive with an accession number, DRA000915. Contaminant reads and low quality reads were removed. Clean reads were used to analyze length distribution and were mapped to the human genome by SOAP [18]. Only the sequences with unique matches were retained for further analysis. Sequences with a perfect match were annotated by comparisons with sequences in the NCBI GenBank (http://www.ncbi.nlm.nih.gov/GenBank/) [19] and Rfam (http://rfam.sanger.ac.uk/) databases [20] to identify noncoding RNAs including rRNAs, tRNAs, snRNAs, snoRNAs, scRNAs, etc. The known miRNAs were annotated by aligning to miRBase 18.0 (http://www.mirbase.org/index.shtml). The remaining reads that cannot be annotated were used to predict novel miRNAs by Mireap (http://sourceforge.net/projects/mireap/).

### Differential expression analysis of miRNAs

For differential expression analysis, the expression of miRNAs from the two libraries were first normalized as follows: normalized expression value = actual miRNA count/total count of clean read * 1,000,000. miRNAs from the two samples with normalized reads less than 1 were removed. The expression values were corrected to 0.01 if the normalized value was found to be 0. The fold change was calculated as a log2 value (treatment/control). The P-value was calculated as follows:
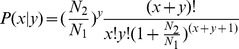


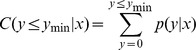


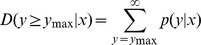



### miRNA target prediction and functional annotation

The target genes of differentially expressed miRNAs were predicted using TargetScan software (http://www.targetscan.org/) [11] and PITA (http://genie.weizmann.ac.il/pubs/mir07/mir07_data.html) [21]. we performed Gene Ontology (GO) [22] enrichment analysis of the target genes to investigate the functional distribution of differentially expressed miRNAs from lung cancer with and without bone metastasis. The web based software WebGestalt [23] was used to perform this analysis. In addition, we also performed the Kyoto Encyclopedia of Genes and Genomes (KEGG) [24] enrichment analysis for identifying the biological pathways associated with bone metastasis.

### Quantitative real-time PCR

Quantitative real-time PCR (qRT-PCR) was performed to validate the results obtained from the high-throughput sequencing. For each sample, cDNA was reverse transcribed from total RNAs by the Ncode ™VILO™ cDNA Synthesis Kit. RT-PCR was performed by the DNA Engine OpticonTM 2 PCR instrument according to the manufacturer's protocol. As template for qRT-PCR validation, 1.33 µL of cDNA was used. U6 snRNA was used as an internal control.

## Results

### Analysis of sRNAs

**Figure 1:**
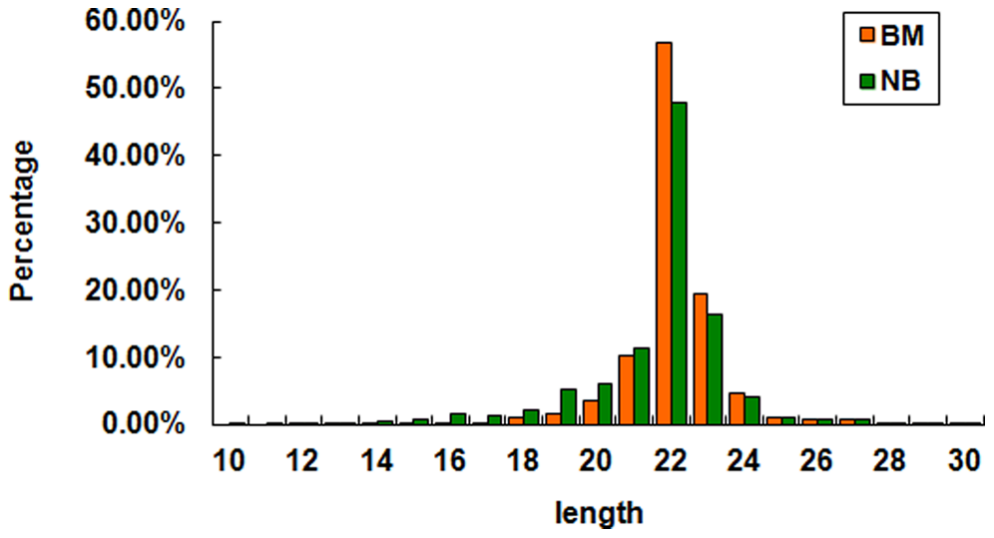
Length distribution and abundance of the sRNAs from BM and NM samples. sRNA reads with a length of 22 nt were the most abundant, which accounting for about half of total reads, then followed by 23 and 21 nt reads.

For identification of the differentially expressed miRNAs, we constructed two sRNA libraries from blood samples of lung adenocarcinoma patients with and without bone metastasis. A total of 24.6 million (bone metastasis: BM) and 25.1 million (non-bone metastasis: NM) raw reads were generated from the two libraries respectively. As seen in Figure 1, most reads had lengths of 18–25 nt. Reads with a length of 22 nt were the most abundant, followed by 23 and 21 nt reads, which correspond to the average length of miRNAs. sRNAs of 20–24 nt in length accounted for 94.7% (BM) and 85.7% (NM) of the total number of sRNA reads. After removing contaminant reads, we obtained clean reads of 12–30 nt in length that accounted for 98.6% (BM) and 94.3% (NM) of total reads.

**Figure 2:**
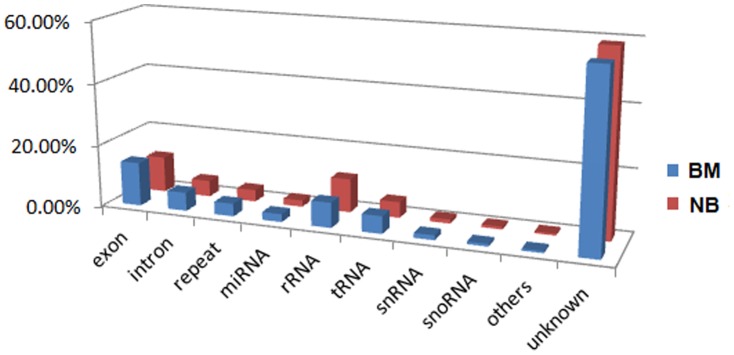
Distribution of different sRNA categories in BM and NM libraries. **s**equenced sRNAs were annotated and classified as: intron, extron, rRNAs, tRNAs, snRNAs, snoRNAs, miRNAs, etc.

sRNAs were then mapped to the reference sequences from GenBank (http://www.ncbi.nlm.nih.gov/Genbank), Rfam (http://rfam.sanger.ac.uk), miRBase (http://www.mirbase.org/) and the human genome. Sequenced sRNAs were annotated as: introns, extronss, rRNAs, tRNAs, snRNAs, snoRNAs, miRNAs, repeat, etc. As shown in Figure 2, the proportion of known miRNAs decreased from 2.55% in samples of bone metastasis to 2.06% in samples of non-metastasis, which implies that some miRNAs play important roles in the bone metastasis of lung cancer. In contrast, the proportion of unknown sRNAs increased from 56.7% to 58.8% in samples with bone metastasis, suggesting that unknown metastasis-related sRNAs remain to be identified.

### Identification of novel miRNAs in the human genome

Due to the rapid development of high-throughput small RNA sequencing methods, an increasing number of miRNAs involved in plant and animal development have been identified. Here, we used the miRNA prediction software Mireap (http://sourceforge.net/projects/mireap/), which is based on the calculation of characteristic hairpin structures of pre-miRNAs and Dicer digestion sites, to predict novel miRNAs in the small libraries. We identified 107 novel miRNA sequences from both libraries, with 57 and 61 novel miRNAs in the sample of BM and NM respectively. Twenty-eight novel miRNAs were expressed in both of the samples (Table S1). Most of the novel miRNA had a length of 21 nt and had uracil (U) as their first nucleotide. The average precursor length was 130 nt with a minimal folding free energy varying from −23.0 to −89.6 kcal/mol, and an average of −48.96 kcal/mol.

### Differential expression analysis of miRNAs of lung cancer with and without bone metastasis

Based on the high-throughput sequencing of small RNAs, we performed differential expression analysis of miRNAs in the two libraries from BM and NM samples. We first removed the miRNAs that have extremely low expression levels (normalization reads <1). miRNAs were considered to be significantly up-regulated or down-regulated if the fold-changes were greater than 2 or less than −2, respectively, with a P-value of less than 0.05. For the known miRNAs in the human genome, 28 miRNAs were identified to be differentially expressed, with 21 up-regulated and 7 down-regulated. As shown in Table 1, hsa-miR-4473 is the most down-regulated miRNA compared with the control, representing approximately a four-fold change. In contrast, the hsa-miR-125b and hsa-miR-548 are the most up-regulated, representing approximately a seven-fold change. For novel miRNAs, 30 miRNAs were identified to be differentially expressed, with 18 were up-regulated and 12 down-regulated (Table S2).

**Table 1:** Summary of differentially expressed miRNAs.

miR name	fold-change	p-value	Status
hsa-miR-4773	−3.93085912	0.000279664	down-regulated
hsa-miR-365a-5p	−3.626016	1.63E-08	down-regulated
hsa-miR-196a-5p	−3.4089189	3.49E-09	down-regulated
hsa-miR-32-3p	−2.70841799	0.004499761	down-regulated
hsa-miR-187-3p	−2.53857823	0.001371206	down-regulated
hsa-miR-34a-5p	−2.49247468	7.61E-06	down-regulated
hsa-miR-21-3p	−2.27534465	6.12E-12	down-regulated
hsa-miR-377-5p	2.09915672	0.012601836	up-regulated
hsa-miR-296-3p	2.15683224	4.91E-05	up-regulated
hsa-miR-27b-3p	2.20970751	0	up-regulated
hsa-miR-106a-5p	2.23030507	1.26E-06	up-regulated
hsa-miR-125a-5p	2.30633203	1.22E-64	up-regulated
hsa-miR-203	2.46128988	0.011805279	up-regulated
hsa-miR-125b-5p	2.63566213	1.02E-13	up-regulated
hsa-miR-550a-3p	2.68366462	0.004077813	up-regulated
hsa-miR-4433-3p	2.69936903	0	up-regulated
hsa-miR-320d	2.77709983	5.31E-18	up-regulated
hsa-miR-320c	2.84603218	1.38E-149	up-regulated
hsa-miR-4286	3.01961409	4.51E-27	up-regulated
hsa-miR-378d	3.26900343	1.42E-08	up-regulated
hsa-miR-199b-5p	3.31684975	0	up-regulated
hsa-miR-491-5p	3.46128988	0.003037459	up-regulated
hsa-miR-550a-3-5p	3.63125615	2.58E-06	up-regulated
hsa-miR-550a-5p	3.63125615	2.58E-06	up-regulated
hsa-miR-18b-5p	3.9638412	0.000155776	up-regulated
hsa-miR-4454	4.12428748	4.66E-05	up-regulated
hsa-miR-125b-2-3p	7.08693234	0.000114317	up-regulated
hsa-miR-548av-5p	7.27965701	3.11E-05	up-regulated

### qRT-PCR validation of differentially expressed miRNAs

**Figure 3:**
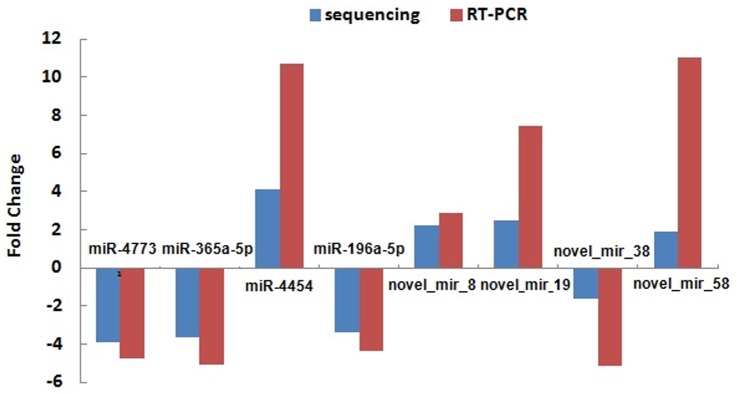
Comparison between high-throughput sequencing data and Quantitative real-time PCR. Known miRNAs including hsa-miR-4773, hsa-miR-365a-5p, hsa-miR-4454 and hsa-miR-196a-5p, and novel miRNAs including novel_mir_8, novel_mir_19, novel_mir_38 and novel_mir_58 were used to perform expression analysis by quantitative real-time PCR. Comparison between high-throughput sequencing and qRT-PCR results indicated that there is good correlation between two platforms.

To validate the miRNA microarray data, the level of randomly selected miRNAs (hsa-miR-4773, hsa-miR-365a, hsa-miR-196a-5p, hsa-miR-4454, novel-mir-38, novel-mir-8, novel-mir-58, and novel-mir-19) were quantified by quantitative RT-PCR. The RT-PCR results for miRNAs and mRNAs presented in Figure 3 demonstrate a very good correspondence between the two platforms.

### miRNA target prediction and functional annotation

We used the online software TargetScan with default parameters combined with PITA to predict the target genes of the differentially expressed miRNAs. A total of 797 target genes were identified for the known miRNAs. To describe the network of miRNAs and target genes involved in bony metastasis of lung adenocarcinoma, we constructed a regulatory network diagram. As shown in Figure S1, a total of 825 nodes and 863 edges were included in this network. Within this network, ATXN1, CHES1, BCL2, SFRS1 and NUFIP2 demonstrated the highest connectivity, which were regulated by 10, 9, 9, 7 and 6 miRNAs respectively.

**Figure 4:**
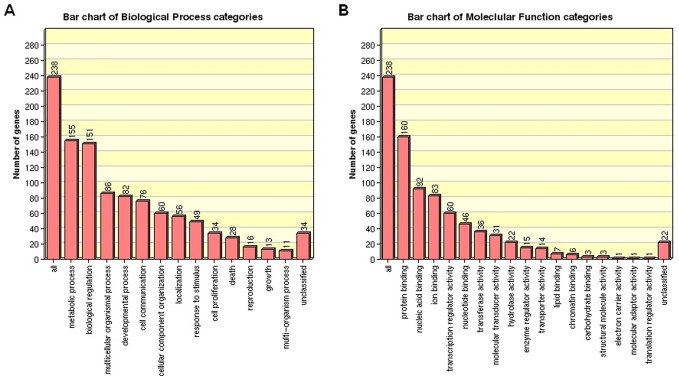
categories and distribution of the Gene Ontology terms of predicted miRNA targets. (A). biological process terms are enriched in metabolic processes, biological regulation and cell communication processes. (B). molecular function terms are enriched in protein binding, nucleic acid binding and ion binding, etc.

To gain insights into the biological implications of differentially expressed miRNAs, we assessed miRNA target genes within the regulatory network for enrichment in Gene Ontology (GO) categories. Gene ontology is one of the most useful methods for functional annotation and classification of genes and gene products. It provides a common descriptive framework for analyze the microarray data. GO categories are organized into three groups: biological process, cellular component, and molecular function. The three groups characterize different aspects of a gene's function and are thus examined separately in our analysis. In this study, a total of 296 miRNA target gene with more than four connectivities were submitted into WebGestalt. Hence, GO analyses were done on 271 genes that have Entrez Gene ID. Genes that showed a nominal significance of P<0.01 were selected and tested against the background set of all genes with GO annotations. We found several GO terms significantly enriched (FDR<0.01) for these miRNA target genes, among which were GO processes related to their biological functions. All enriched GO terms are presented in Figure 4. We found a significant over-representation of metabolic processes, biological regulation and cell communication processes for the biological process and protein binding, nucleic acid binding and ion binding for the molecular function. To further discover the biological pathways affected in bone metastasis, we also performed the KEGG pathway enrichment analysis for the differentially expressed genes. As shown in Table 2, the top canonical pathways of differentially expressed mRNAs targets include pathways in cancer (P = 1.23e-11), MAPK signaling pathway (P = 2.46e-15), small cell lung adenocarcinoma (P = 2.797e-06) and prostate cancer (P = 4.35e-07).

**Table 2:** This table lists the enriched KEGG pathways, number of differentially expressed genes, enriched gene id and adjusted p value from Hyper-geometric test.

KEGG pathway	number of genes	Entrnz gene ID	P value
Pathways in cancer	18	8322 1871 324 598 867 2113 5156 5925 998 10000 4286 3479 596 861 3815 5594 6774 6789	1.23E-11
MAPK signaling pathway	13	55970 4149 1848 23162 5578 5495 5156 5606 3727 3725 5880 6788 5062	2.46E-15
Wnt signaling pathway	20	6423 1460 5578 3725 595 56998 5880 4775 6424 896 8313 166336 2932 5527 27101 4776 9475 1487 818 5567	5.77E-09
Small cell lung cancer	12	595 10401 5743 5925 4149 207 1284 10000 331 1163 1027 3918	2.79E-06
Prostate cancer	8	596 5925 9586 5594 5156 10000 1871 3479	4.35E-07
TGF-beta signaling pathway	8	3398 6198 3400 90 659 5594 57154 4092	4.35E-07
Glioma	7	808 5925 5594 5156 10000 1871 3479	5.62E-07
NF-KappaB signaling pathway	14	595 3574 10401 163702 161742 207 10254 6778 10000 896 10379 81848 3977 9655	4.36E-05
Insulin signaling pathway	9	7248 10000 122809 808 2872 6198 867 1977 5594	5.62E-07
Pancreatic cancer	7	5925 598 998 6774 5594 10000 1871	8.25E-07
Melanoma	7	5925 5594 5156 10000 4286 1871 3479	8.25E-07
Chronic myeloid leukemia	7	5925 861 598 867 5594 10000 1871	9.55E-07
mTOR signaling pathway	6	7248 6198 5594 1977 10000 3479	1.89E-06
Acute myeloid leukemia	6	6198 861 6774 5594 3815 10000	3.67E-06

## Discussion

Bone metastasis is the most commonly observed type of metastasis for lung adenocarcinoma and is the major cause of the poor outcome associated with this disease. It refers to lung cancer cells that spread to bone through the bloodstream or lymphatics. However, the underlying causes of bone metastasis of lung adenocarcinoma remain largely unknown to-date. Thus, understanding the pathogenesis of bone metastasis of lung adenocarcinoma could have the important implications for drug development and treatment for this disease. A number of biomarkers related to the lung adenocarcinoma have been identified by genome wide expression profiling studies. Gene expression signatures combined with basic clinical covariates (stage, age, sex) can be used to predict overall survival in lung cancer subjects [25]. miRNA expression profiling was also used to classify different samples from lung cancer compared with in non-tumorous samples. For example, when Ma *et al.* generated both mRNA and miRNA expression data from non-small cell lung cancer tissues, a total of 25 miRNAs exhibited higher expression and 24 miRNAs demonstrated lower expression compared to non-tumorous samples; these critical genes may play an important role in cancer development and have great potential to be used as biomarkers for diagnosing lung adenocarcinoma [26]. These findings have dramatically improved our knowledge of the pathophysiology of lung adenocarcinoma. However, there is very little reported on the miRNA expression profiles of lung adenocarcinoma bone metastasis.

Blood is thought to be ideal for sample collection as it can be obtained easily in an less invasive manner than from pathological tissues directly. Recent studies indicated that serum miRNAs may serve as a novel class of biomarkers for accurate diagnosis and prognosis. So far, analysis of circulating microRNAs in cases of bone metastasis of lung adenocarcinoma have not been reported. In this study, we collected blood samples from lung adenocarcinoma patients either with or without bone metastasis and performed miRNA expression profiling analysis using high-throughput sequencing. A total of 32 differentially expressed miRNA could classify these different samples, which consists of 7 up-regulated and down-regulated respectively. In addition, some novel miRNAs were also identified in both samples. This result demonstrates that miRNA expression signatures may tightly correlate with bony metastasis and the survival of patients with lung adenocarcinoma. Actually, some of these miRNAs have previously been reported to be closely linked with lung adenocarcinoma metastasis. For example, Wang et al. indicated that has-miR-183 is an inhibiting factor for lung adenocarcinoma metastasis. has-miR-183 mainly functions to regulate lung adenocarcinoma metastasis by regulating the expression of Ezrin and other metastasis-related genes. Loss of expression of this miRNA could promote the metastasis process of lung adenocarcinoma [27]. Expression profiling analysis by high-throughput sequencing and qRT-PCR in this study confirmed that there is a differential expression of both miR-183-3p and miR-183-5p in the samples of lung cancer with and without metastasis, and a decreased expression was observed in the metastatic sample. Another miRNA, the miR-574-5p has been reported to suppress Qki6/7. Qki6/7 functions to regulate the ß-catenin/Wnt pathway, which is involved in the occurrence and development of colorectal cancer [28]. This study also confirmed that miR-574-5p is present at increased levels in metastatic samples of lung adenocarcinoma compared to the non-metastatic samples. Thus, the expression of miR-574-5p seems to be closely related to the development and transfer of lung adenocarcinoma.

In addition, the misexpression of other miRNAs has also been correlated with cancer development. For example, miR-26 was identified to promote growth and invasion in non-small cell lung adenocarcinoma by repressing the tumor suppressor PTEN [29]. The tumor-suppressor protein p53 can activate the miRNA-34 family, which is a tumor-suppressor gene [30]. Interestingly, the miR-34 family was identified to inhibit the osteoblast proliferation and differentiation by targeting SATB2 and other cell cycle control factors, thus it will be valuable to study whether the dysfunction of this miRNA regulated pathways could affect the bone metastasis of lung adenocarcinoma [31]. The decreased expression of this family may contribute to certain cancer types, including non-small cell lung cancer. miR-320 family was identified to be differentially expressed in many cancer types, including breast cancer, colorectal cancer, prostate cancer and retinoblastoma [32], [33], [34]. In addition, another up-regulated miRNA, the miR-296 was predicted to regulate the tumor suppressor Sirt6. Sirt6 is known to regulates aerobic glycolysis in cancer cells and the down-regulation of Sirt6 is observed in several human cancers [35]. Theoretically, the up-regulation of miR-296 will inhibit the expression of Sirt6, although significant change of Sirt6 expression is to be further confirmed, especially in the bone metastasis of long adenocarcinoma.

Further bioinformatics analysis could help us to investigate the roles that the deregulated miRNAs played in the bone metastasis of lung adenocarcinoma. Gene Ontology and KEGG pathway enrichment analyses were used to interpret the biological functions of the miRNA targets. GO analysis of miRNA targets in this study indicated that the miRNAs play important roles in regulation of gene expression, metabolic processes, cell proliferation, cellular component organization and apoptosis. For the KEGG pathway enrichment analysis, the top over-represented pathways identified included the MAPK signaling pathway and pathways involved in cancer. These results presented in this study illustrate some of the underlying biological processes that may be involved in bone metastasis.

The disadvantage of this strategy may be in that only the blood samples were used for miRNA expression profiling analysis. Although blood is thought to be ideal for sample collection as it can be obtained easily in an less invasive manner, some miRNAs could be missed in blood but expressed in tumors' tissues. Thus a detailed follow-up study is needed. In summary, our study indicates that by integrating the miRNA expression data and bioinformatics analysis, a miRNA-regulated network that may involved in the bone metastasis of lung adenocarcinoma was identified. Although detailed follow-up study is still needed, our results may provides an important contribution to characterize the role of specific miRNAs in pathogenesis of bone metastasis and thereby helping with the improvement of diagnosis and treatment of lung adenocarcinoma.

## Supporting Information

Figure S1 **miRNA-mediated regulatory network in the bone metastasis of lung cancer.** The red nodes represent the miRNAs and the green nodes represent their targets.
(TIF)

Table S1 **Lists of newly identified miRNAs in BM and NM samples.** A total of 107 novel miRNAs were identified in BM and NM samples.
(DOC)

Table S2 **Summary of newly miRNAs that are differentially expressed in BM and NM samples.** A total of 30 miRNAs were identified to be differentially expressed, and 18 were up-regulated and 12 were down-regulated.
(DOC)
