# Supplementary figures and images for: Genome-Wide Identification of Bone Metastasis-Related MicroRNAs in Lung Adenocarcinoma by High-Throughput Sequencing
Source: PLoS One. 2013 Apr 8;8(4):e61212. doi: 10.1371/journal.pone.0061212 (PMC3620207; doi:10.1371/journal.pone.0061212)

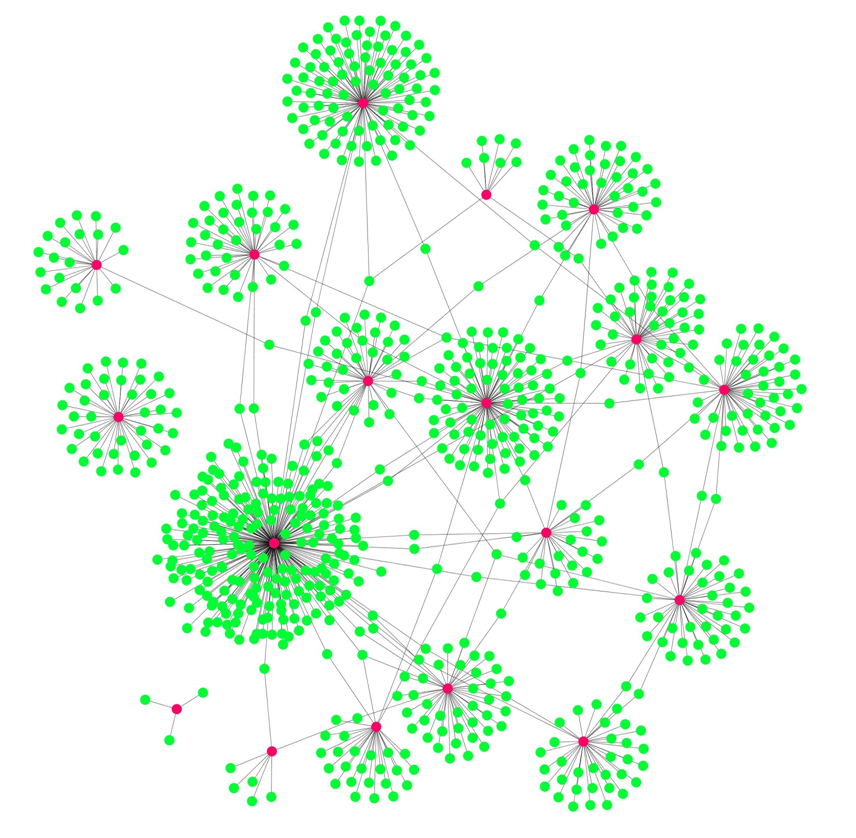

Supplement: Figure S1 — miRNA-mediated regulatory network in the bone metastasis of lung cancer. The red nodes represent the miRNAs and the green nodes represent their targets. (TIF) [file pone.0061212.s001.tif]
