# Supplementary material for: Genome-Wide Identification of Bone Metastasis-Related MicroRNAs in Lung Adenocarcinoma by High-Throughput Sequencing
Source: PLoS One. 2013 Apr 8;8(4):e61212. doi: 10.1371/journal.pone.0061212 (PMC3620207; doi:10.1371/journal.pone.0061212)
Supplement: Table S1 — Lists of newly identified miRNAs in BM and NM samples. A total of 107 novel miRNAs were identified in BM and NM samples. (DOC) [file pone.0061212.s002.doc]

**Table S1:** List of newly identified miRNAs in BM and NM samples.

| Novel miRNA name | miRNA sequence | miRNA count in BM | miRNA count in NM |
| --- | --- | --- | --- |
| novel_mir_1 | TCGACTTGCTCGGGCCCGGCT | 6 | 13 |
| novel_mir_10 | CTGGAGGGAGGGAGGAAGGGC | 5 | 0 |
| novel_mir_100 | TCTTTATATGTACTGGAGCCCGA | 0 | 7 |
| novel_mir_101 | TGGGCGGCCACTTGACATCCTC | 0 | 20 |
| novel_mir_102 | TCCTGTACTGAGCTGCCCCGAGC | 0 | 7 |
| novel_mir_103 | TGAGTGTGTGTGTGTGAGTGTGA | 0 | 8 |
| novel_mir_104 | AGGGCCGAAGGGTGGAAGCT | 0 | 19 |
| novel_mir_105 | ATCTGAGAGACTGAGATGGCA | 0 | 5 |
| novel_mir_106 | CCGGGTGTAGGGCGGGCGGG | 0 | 6 |
| novel_mir_107 | TTGGGAGAGAAGAGAAGAGTGCT | 0 | 7 |
| novel_mir_11 | GCGGCCGGGCTGGGCGTGTGTG | 6 | 0 |
| novel_mir_12 | TTGTGGAAACAATGGTACGGCA | 5 | 12 |
| novel_mir_13 | GGCTCGTGGCCTGCAGTGGATG | 7 | 11 |
| novel_mir_14 | TGACACTGTGGGGAACGTGCA | 12 | 33 |
| novel_mir_15 | CGAGGCTGTCGGCGCGCGCCCAT | 7 | 0 |
| novel_mir_16 | ACTGGCAAAAGGGTTTAGAACT | 27 | 27 |
| novel_mir_17 | TGGGGAGGAAGGCTGGGAGAAG | 5 | 0 |
| novel_mir_18 | CTGGGAGGGGCTGGGTTTGGC | 26 | 0 |
| novel_mir_19 | GAAGCAGCGCCTGTCGCAACT | 10 | 61 |
| novel_mir_2 | AAATGAATCATGTTGGGCCTG | 7 | 0 |
| novel_mir_20 | TGGGGCCAGCTTTGGAAGCCTGCA | 7 | 0 |
| novel_mir_21 | TCAGGGGATGGGAGTGACATGGC | 7 | 0 |
| novel_mir_22 | CTTTGGACAGAGAGGGTGTAGGT | 6 | 0 |
| novel_mir_23 | TGTGGGGCAGGGCAAGGGAGCG | 6 | 0 |
| novel_mir_24 | CTGGGAGGCGGTCGGTTCTGA | 7 | 0 |
| novel_mir_25 | CCGCGGGGGAGGGGAGGGGAGGG | 7 | 7 |
| novel_mir_26 | TTGGAGGGTGTGGAAGACAT | 12 | 10 |
| novel_mir_27 | TGGGGGACGAGAGAGTGACAGTA | 15 | 19 |
| novel_mir_28 | TCGGCGCGGGCGGGCAGGGCC | 19 | 0 |
| novel_mir_29 | TGGGCTAGTGAACGCGGCGAAGT | 8 | 0 |
| novel_mir_3 | GAGGACCCTGCAGGAATGGACG | 10 | 0 |
| novel_mir_30 | TTGAGGGGAGAATGAGGTGGAGA | 62 | 0 |
| novel_mir_31 | AGCAGAGACGTTGGAACTGGGCT | 9 | 0 |
| novel_mir_32 | TGGCGTGGAAGATGCTGCCA | 5 | 0 |
| novel_mir_33 | CTCTGCGGGTGCGGAGCGAGCTTT | 7 | 0 |
| novel_mir_34 | TGCCCGGCGGTGTGCGGCCACA | 6 | 0 |
| novel_mir_35 | AGGGGCTGGGGTTTCAGGTT | 19 | 38 |
| novel_mir_36 | ACTGCTGCAGATGGAAAAGTT | 9 | 22 |
| novel_mir_37 | GGAGGAACCTTGGAGCTTCGGCA | 231 | 209 |
| novel_mir_38 | CCGGGAGGGCAGTGGAGGCGTG | 17 | 6 |
| novel_mir_39 | AGAGGAGGGTGGAGCAAGTGGT | 84 | 96 |
| novel_mir_4 | TGGAGGGTGAGGAGTGGCTAT | 28 | 30 |
| novel_mir_40 | ATGGGGACAGGGATCAGCATGGCA | 8 | 0 |
| novel_mir_41 | TGTGAGGAAGAAGAGGATGGAA | 10 | 0 |
| novel_mir_42 | GAGGGAACAGGGGCAGACTTCT | 5 | 6 |
| novel_mir_43 | TTGGAATGGGTGGTTGGGGAA | 7 | 6 |
| novel_mir_44 | CGGAGTGGAGCGGAGCGGGCT | 10 | 0 |
| novel_mir_45 | AAAAAGATTGAGGCCATGGTA | 7 | 15 |
| novel_mir_46 | TGGGGAGGTGTGGAGTCAGCAT | 17 | 21 |
| novel_mir_47 | AAAGGGGACAGCTCACAGGATT | 6 | 0 |
| novel_mir_48 | GACAATTGTTGATCTTGGGCCT | 5 | 0 |
| novel_mir_49 | CGAGAGGAGGAGGAGGAGGCGG | 10 | 0 |
| novel_mir_5 | AGGGGCGCGGCCCAGGAGCTCA | 5 | 0 |
| novel_mir_50 | GGACTGACCGACGGGCGGGCGC | 5 | 0 |
| novel_mir_51 | GATGAGGAGGATGAGGAGGATG | 16 | 59 |
| novel_mir_52 | TCGGGCGGGAGTGGTGGCTTTT | 496 | 531 |
| novel_mir_53 | AACAAGAGAGCAGAACGAGGTT | 18 | 0 |
| novel_mir_54 | TGGGCAGGGGCTTATTGTAGGAGT | 92 | 0 |
| novel_mir_55 | CCGGGCGGGCGAGGAGCGGG | 10 | 13 |
| novel_mir_56 | GGGGCTGGGGGCGCAGGTCG | 9 | 15 |
| novel_mir_57 | GAGGCGACGCGGTGACTGGA | 6 | 0 |
| novel_mir_58 | TGAGCACCCCAGGACCTGCGCT | 5 | 20 |
| novel_mir_59 | TGGGCGGCCACTTGACATCCTCT | 9 | 0 |
| novel_mir_6 | GAGAGCTCCGACTGCAGCTGC | 5 | 10 |
| novel_mir_60 | TCTTTTTGTGTTTTTGAGCGTCTA | 5 | 0 |
| novel_mir_61 | CATGGCACTGGAGTAGAGCAT | 10 | 0 |
| novel_mir_62 | CCCTGGGGTTCTGAGGACATG | 367 | 435 |
| novel_mir_63 | GCGGCGGCAGGTGTAGAGGAG | 12 | 21 |
| novel_mir_64 | TCTGTGGGAGGTGAGACGACG | 15 | 0 |
| novel_mir_65 | AACTTTGGAATGTGGTAGGGTA | 6 | 0 |
| novel_mir_66 | AGGCGAAGTCAGAGAGCTCT | 0 | 6 |
| novel_mir_67 | ACCTGGTGGCTGGCAGGCACT | 0 | 7 |
| novel_mir_68 | AATGAATCATGTTGGGCCTG | 0 | 23 |
| novel_mir_69 | TCTGGAGACTGGGAGTGTTTATT | 0 | 5 |
| novel_mir_7 | TAGAGAGGGGAAGGATGTGATGT | 10 | 0 |
| novel_mir_70 | TCAGGGAGAAAGAAGGGTTATT | 0 | 21 |
| novel_mir_71 | TCTGGAGCACAGACGTCTGGGA | 0 | 9 |
| novel_mir_72 | GACGCGCCCTCCCGCTCCGAG | 0 | 5 |
| novel_mir_73 | CTGGGATTTGGAGTGGCAGCT | 0 | 10 |
| novel_mir_74 | CGGGGTGGAGCGGGAGGCGTC | 0 | 14 |
| novel_mir_75 | CGCGAGGCTGTCGGCGCGCGCC | 0 | 10 |
| novel_mir_76 | TCACGTCTGCGGCTGTCACGT | 0 | 9 |
| novel_mir_77 | TGCGGGTGGGAAAGAGGTGCAGT | 0 | 23 |
| novel_mir_78 | AGCAGGCAGGGATTGGGAGCACT | 0 | 5 |
| novel_mir_79 | TGGCGGGGGTAGAGCTGGCTGC | 0 | 9 |
| novel_mir_8 | AGCGGAGGGCGGAGGGGAGGGGC | 8 | 40 |
| novel_mir_80 | TGGGGCCAGCTTTGGAAGCCTGC | 0 | 9 |
| novel_mir_81 | CAGGGCCGGGGCCGGGACGGG | 0 | 7 |
| novel_mir_82 | GGAGGAGGAGGAGGAGGATAA | 0 | 21 |
| novel_mir_83 | TCCGGATCCGGCTCCGCGCCTCA | 0 | 6 |
| novel_mir_84 | CGGCAGCCGCCGCTCGGGCAA | 0 | 7 |
| novel_mir_85 | TCAGGGGATGGGAGTGACATG | 0 | 7 |
| novel_mir_86 | TTGAGGGGAGAATGAGGTGGAG | 0 | 74 |
| novel_mir_87 | AGCAGAGACGTTGGAACTGGG | 0 | 15 |
| novel_mir_88 | AGGTAGAAGGAGAGAAAAGGAGG | 0 | 7 |
| novel_mir_89 | CCGGCTGTCCAAGAAGAGGG | 0 | 144 |
| novel_mir_9 | TTGTTGAACTAGGGCAGGAT | 5 | 0 |
| novel_mir_90 | ATGGGGACAGGGATCAGCATGG | 0 | 11 |
| novel_mir_91 | TGTGAGGAAGAAGAGGATGG | 0 | 11 |
| novel_mir_92 | GGACGAAATCCAAGCGCAGCT | 0 | 7 |
| novel_mir_93 | TAGGGTTAAGAGTGGGGAGA | 0 | 6 |
| novel_mir_94 | ATGCTTAGATCTCCCCACTGGTA | 0 | 11 |
| novel_mir_95 | ATGGGTTGTAGGTGGATCAGA | 0 | 6 |
| novel_mir_96 | AGGGGAACGTGAGGAGAGCTGCG | 0 | 6 |
| novel_mir_97 | GAGAATATGGGAGTCTGTGGCT | 0 | 14 |
| novel_mir_98 | ACAAGAGAGCAGAACGAGGTT | 0 | 15 |
| novel_mir_99 | CCCCAGGGATGGAGAAAGGG | 0 | 16 |
