# Supplementary material for: Genome-Wide Identification of Bone Metastasis-Related MicroRNAs in Lung Adenocarcinoma by High-Throughput Sequencing
Source: PLoS One. 2013 Apr 8;8(4):e61212. doi: 10.1371/journal.pone.0061212 (PMC3620207; doi:10.1371/journal.pone.0061212)
Supplement: Table S2 — Summary of newly miRNAs that are differentially expressed in BM and NM samples. A total of 30 miRNAs were identified to be differentially expressed, and 18 were up-regulated and 12 were down-regulated. (DOC) [file pone.0061212.s003.doc]

**Table S2:** Summary of novel miRNAs that are differentially expressed in BM and NM samples.

| **miR-name** | **BM expressed** | **NM expressed** | **fold-change** | **p-value** | **Status** |
| --- | --- | --- | --- | --- | --- |
| novel_mir_54 | 92 | 0 | -9.92653315 | 3.48E-30 | downregulated |
| novel_mir_30 | 62 | 0 | -9.35715612 | 1.39E-20 | downregulated |
| novel_mir_18 | 26 | 0 | -8.10339273 | 4.58E-09 | downregulated |
| novel_mir_28 | 19 | 0 | -7.65090812 | 7.96E-07 | downregulated |
| novel_mir_53 | 18 | 0 | -7.57288968 | 1.66E-06 | downregulated |
| novel_mir_64 | 15 | 0 | -7.30988557 | 1.52E-05 | downregulated |
| novel_mir_3 | 10 | 0 | -6.72492307 | 0.000604109 | downregulated |
| novel_mir_41 | 10 | 0 | -6.72492307 | 0.000604109 | downregulated |
| novel_mir_44 | 10 | 0 | -6.72492307 | 0.000604109 | downregulated |
| novel_mir_49 | 10 | 0 | -6.72492307 | 0.000604109 | downregulated |
| novel_mir_61 | 10 | 0 | -6.72492307 | 0.000604109 | downregulated |
| novel_mir_7 | 10 | 0 | -6.72492307 | 0.000604109 | downregulated |
| novel_mir_8 | 8 | 40 | 2.19862118 | 7.85E-06 | upregulated |
| novel_mir_19 | 10 | 61 | 2.4854317 | 2.36E-09 | upregulated |
| novel_mir_90 | 0 | 11 | 6.73903798 | 0.00080667 | upregulated |
| novel_mir_91 | 0 | 11 | 6.73903798 | 0.00080667 | upregulated |
| novel_mir_94 | 0 | 11 | 6.73903798 | 0.00080667 | upregulated |
| novel_mir_74 | 0 | 14 | 7.08693234 | 0.000114317 | upregulated |
| novel_mir_97 | 0 | 14 | 7.08693234 | 0.000114317 | upregulated |
| novel_mir_87 | 0 | 15 | 7.18655998 | 5.96E-05 | upregulated |
| novel_mir_98 | 0 | 15 | 7.18655998 | 5.96E-05 | upregulated |
| novel_mir_99 | 0 | 16 | 7.27965701 | 3.11E-05 | upregulated |
| novel_mir_104 | 0 | 19 | 7.5275552 | 4.40E-06 | upregulated |
| novel_mir_101 | 0 | 20 | 7.60154796 | 2.30E-06 | upregulated |
| novel_mir_70 | 0 | 21 | 7.67193021 | 1.20E-06 | upregulated |
| novel_mir_82 | 0 | 21 | 7.67193021 | 1.20E-06 | upregulated |
| novel_mir_68 | 0 | 23 | 7.80316244 | 3.25E-07 | upregulated |
| novel_mir_77 | 0 | 23 | 7.80316244 | 3.25E-07 | upregulated |
| novel_mir_86 | 0 | 74 | 9.48908528 | 1.22E-21 | upregulated |
| novel_mir_89 | 0 | 144 | 10.44955106 | 1.93E-41 | upregulated |
